# Food habits, physical activities and sedentary lifestyles of eutrophic and obese school children: a case–control study

**DOI:** 10.1186/s12889-015-1491-1

**Published:** 2015-02-11

**Authors:** Jenny Vilchis-Gil, Marcia Galván-Portillo, Miguel Klünder-Klünder, Miguel Cruz, Samuel Flores-Huerta

**Affiliations:** Community Health Research Department, Hospital Infantil de México Federico Gomez, Ministry of Health (SSA), Mexico City, Mexico; Center for Population Health Research, National Institute of Public Health, Cuernavaca, Morelos Mexico; Medical Research Unit in Biochemistry, UMAE Bernardo Sepulveda, IMSS, Mexico City, Mexico

**Keywords:** Food habits, Physical activity, Sedentary lifestyle, Obesity, Children

## Abstract

**Background:**

Civilization has produced lifestyle changes; currently, people ingest more calories than are expended, resulting in obesity. This study assessed the association between dietary habits, physical activities, and sedentary behaviors and the risk of obesity in schoolchildren in Mexico City.

**Methods:**

Of 1,441 children (6–12 years old) screened in elementary schools, 202 obese (BMI ≥95^th^ pc) and 200 normal-weight children (BMI 25^th^- 75^th^ pc), as defined by the 2000 CDC criteria, were included in a case–control study. The children’s eating, physical activity and sedentary lifestyle habits were recorded using validated questionnaires. The quantity and quality of the foods were obtained, and the energy that was expended was transformed into METs. Sedentary behavior was assessed in hours. Logistic regression models were used to determine the risks of certain habits and their association with obesity.

**Results:**

Obese children ingested around of 270 Kcal less than eutrophic children. However, compared with the eutrophic children, obese children had significantly worse lifestyle habits; the children with healthy dietary habits (eating breakfast at home, bringing a school lunch, and not bringing money to purchase food) had a lower risk of obesity (OR 0.59, CI 0.46; 0.75). The quality of the eaten food was associated with a risk of obesity. Consuming fruit demonstrated an inverse association with risk of obesity (*p Trend* = 0.01); consumption of sweetened beverages (*p Trend* < 0.04) and refined carbohydrates with added fat (*p Trend* = 0.002) were associated with an increased risk of obesity. Children who were more physically active at school had an OR of 0.37 (CI 0.16; 0.89), those who had 3–4 televisions at home had an OR of 2.13 (CI 1.20; 3.78), and the risk of developing obesity was independent of caloric intake.

**Conclusions:**

Poorer eating habits as well as less physical activity were associated with the risk of obesity. An obesogenic environment could change if teachers and parents worked together to form healthy food intake and physical activity habits.

## Background

Currently, overweight and obesity represent a public health problem that affects populations of all cultures, socioeconomic classes, and ages, including children [1]. Obesity has been considered to result from lifestyle changes, especially in food consumption, physical activity and sedentary tendencies, because the environment has grown more obesogenic in recent decades. Regarding food habits, there have been the following two opposing tendencies: 1) an increasing consumption of high-caloric industrialized foods and beverages; for instance, Mexican school children obtain approximately 20.7% of their energy from sweetened beverages [2], and 2) a decreasing consumption of natural and healthy foods, such as fruits, vegetables, legumes and whole grains, which is a habit considered to be a risk factor for obesity [2,3].

Additionally, Western societies, including children, display an increased tendency toward sedentary lifestyles, including resting, watching TV, traveling by car to and from school and performing less physical activity [4]. Physical activity has an important role in maintaining a healthy nutritional status, while sedentary lifestyles are a risk factor for obesity and its comorbidities [5].

According to the Mexican National Nutrition Surveys, the combined prevalence of overweight and obesity in schoolchildren increased from 26.9% in 1999 to 34.8% in 2006. Although the 2012 survey indicates that the prevalence of overweight and obesity has not increased over the past 6 years (34.4%) [6], the percentage is one of the highest among school-age children (5–11 years) in Latin America [7]. The recent increase in the problem is likely associated with changes in the lifestyles of families [8]. Therefore, the aim of the present study was to 1) characterize the dietary habits, exercise, and sedentary lifestyles of school children in Mexico City according to eutrophic or obese nutritional status and 2) identify which habits regarding modified foods or exercise styles lead children who live in an obesogenic environment to develop overweight or obesity.

## Methods

### Procedure and participants

From February 2008 to December 2010, a case–control study was conducted in nine middle-class elementary schools in Mexico City, which did not represent all socio-economic groups. The study was approved by the Research, Ethic and Biosecurity Committees of the Hospital Infantil de Mexico Federico Gómez; written informed assent and consent were obtained from participants and their parents, respectively.

Because this report is part of another comprehensive study in which the genes associated with obesity were examined [9], the sample was designed to contain two groups that contrasted in their nutritional status, normal weight or obese children, and overweight children were excluded. The participants in this study were school children between 6 and 12 years of age, including obese (cases) and normal-weight children (controls). The children were considered to be obese when their body mass index (BMI) for age and gender were in the ≥95th percentile, whereas the children were considered to be a normal weight when their BMI was between the 25th and 75th percentiles, as defined by the Centers for Disease Control and Prevention 2000 [10]. Individuals who were overweight (BMI >75th but <95th percentile) or malnourished children (BMI < 25th percentile) were excluded.

### Instruments

The procedures for measuring anthropometric data and gathering information on food habits, food intake, and physical activity/sedentary lifestyles were conducted by two nutritionists and two nurses from our staff, who were trained and standardized in all procedures. Anthropometric measurements were conducted following international protocols that have been described elsewhere [11], and the Frequency of Food Questionnaires (FFQ) were administered and followed a reference manual for dietetic survey [12]. Information was gathered on children’s physical activity/sedentary lifestyles using a previously validated questionnaire that details activities inside and outside of school [13].

### Anthropometric measures

Briefly, the weights and the heights of children were recorded while they wore lightweight clothes and no shoes. Their weights were measured to the nearest 0.1 kg using a digital scale (SECA model-882, SECA Corp., Hamburg, Germany); their heights were measured to the nearest 0.1 cm using a stadiometer (SECA model-225, SECA Corp., Hamburg, Germany). Waist circumference was measured at the end of an exhalation with non-elastic flexible tape (Seca 200) in a standing position at the midpoint between the lower costal border and the iliac crest. In order to decrease any parallax error, participants were asked to climb on an anthropometric box designed for this purpose.

### Questionnaire on dietary habits during the school day

The children and their parents were asked the following three main questions: 1) whether the child had eaten breakfast at home, 2) whether the child had brought his/her lunch to eat at school, and 3) whether the child had brought money to buy food at school. The children’s responses required them to only recall information from the day of the study; however, the parents’ responses involved a recall of information from the previous week. Eating breakfast at home, bringing lunch to school, and not bringing money to purchase food at school were considered to be healthy habits. We did not estimate the amount or the quality of the foods that children brought for lunch.

### Frequency of food consumed

This measure was collected using an adapted version of the semi-quantitative FFQ for information from the previous month. The course of fieldwork in the schools lasted from March 2008 to May 2009, and questionnaires were applied from Monday to Friday; a meeting with the parents was arranged in advance before school activities began. The questionnaire was sent to parents who could not attend, and another appointment was made to administer the questionnaire to their child. As support material, the interviewer used food replicas in order to standardize the types and amounts of the main food groups consumed by the participants.

The questionnaire contained 110 food items classified in 13 groups. Participants’ food intake per day was estimated, and the amount of food consumed was calculated in terms of the units of measurement (e.g., piece, cup, plate, or spoon) and the size of the unit (i.e., small, medium, or large). For the analysis, the frequencies of consumption were calculated in grams or milliliters ingested per day for each of 110 food items. The participants’ daily intake levels of energy, macronutrients, and fiber were calculated using a food composition database from the USDA-SR23 [14] and Mexican food tables that include data regarding traditional Mexican food [15,16]. The percentage of adequate energy and fiber intake were calculated using the recommendations for Mexican populations [17]. The foods were categorized as solid or liquid and then re-categorized according to their cardiovascular risk. The category of foods without cardiovascular risk included fruits, vegetables, cereals and legumes (e.g., corn tortillas, rice, and oats). On the other hand, foods with cardiovascular risk included the following: those food made from refined flour (e.g., white bread, bread rolls, and wheat flour tortillas), dishes with added fat such as “*tamales*” (steamed or fried), “*sopes*” (fried stuffed tortillas), natural fruit juices and industrialized sweetened beverages (e.g., soft drinks, energy drinks, and commercial juices). Finally, the foods were analyzed for their cardiovascular risk according to tertiles of consumption.

### Physical activity and sedentary lifestyle

Inside the school, physical activity was estimated using the number of days and hours that children participated in physical education classes and expressed as the number of hours per week. To estimate the amount of exercise performed outside of school, each child was asked to provide the number of days and times per week that he/she played sports such as soccer, football, basketball, cycling, and swimming or engaged in active free playing. Based on the number of days and hours per day of exercise, the metabolic equivalents (METs), which are multiples of the resting metabolic rates, were estimated. This value corresponds to 3.5 mL O_2_/kg^−1^/min^−1^[18], and the children were grouped into tertiles for the analysis.

Sedentary behavior was assessed based on the following information: 1) the number of television sets at home, 2) the time spent in front of a screen (television, computer, or video games; categorized as ≤2 h/d or >2 h/d), and 3) the type of transportation used to go from home to school on an ordinary weekday. In addition, each participant’s cumulative (daytime and nighttime) sleeping hours were categorized as <9 h/d or ≥9 h/d.

### Data analysis

Tests were performed to identify the differences between the cases and the controls regarding the following variables: socio-demographic and anthropometric data, dietary habits, physical activity and sedentary lifestyles. The groups were compared using an unpaired Student’s t-test for the continuous variables and Pearson’s χ^2^ test for the categorical variables. The differences in the medians of energy and macronutrient intake were evaluated using Mann-Whitney’s U test. To evaluate the association between dietary habits, exercise, and sedentary lifestyle and obesity, odds ratios (ORs) and 95% confidence intervals (CIs) were estimated using multivariate logistic regression models. The first model was built to evaluate the association between obesity (dependent variable) and each one of the independent variables (dietary habits, carbohydrates with or without cardiovascular risk, exercise, sedentary behavior, and number of hours of sleep), adjusting by age, gender, and energy intake. Using this information, another model that best fit the data was used, which included different variables (fruits, vegetables, cereals, fried foods from wheat or corn, natural fruit juice, sweetened commercial beverages, physical activity at school, television sets at home and number of hours of sleep), adjusted by age, gender, energy intake and METs. In both models, the correlations within the schools were considered. Additionally, in both regression models, the first tertile of consuming the different types of foods was considered to be the reference category; the assumptions of the logistic regression models were also examined. A *p* value <0.05 was accepted to be statistically significant. The analysis was performed using the STATA/SE v.11.0 statistical software package (Stata Corporation, College Station, TX, USA).

## Results

After screening 1,441 children, 202 children were classified as obese (cases), and 200 children were classified as normal-weight (controls). The demographic and anthropometric characteristics of these children are shown in Table 1. The children identified as cases were heavier and taller than the controls. Concerning energy intake, the obese children consumed less than normal-weight children (1912 Kcal/d vs 2181 Kcal/d, *p* = 0.002) (Table 2). Consequently, obese children consumed fewer macronutrients, cholesterol, and fiber than controls, although the percentage of the nutrient distribution was similar in both groups.

**Table 1:** **Demographic and anthropometric characteristics of the schoolchildren, cases and controls**

**Characteristics**	**All**	**Obese**	**Normal-weight**	***p*** **-value** ^**a**^
	**Mean ± SD**	**Mean ± SD**	**Mean ± SD**	
*n*	402	202	200	
Age (y)	9.4 ± 1.8	9.5 ± 1.7	9.4 ± 1.8	0.48
Female (%)	44.8	43.1	46.5	0.49
Weight (kg)	38.9 ± 13.4	48 ± 11.9	29.8 ± 7.2	<0.001
Height (cm)	136.3 ± 12.1	139.0 ± 11.4	133.6 ± 12.1	<0.001
BMI (kg/m^2^)	20.4 ± 4.5	24.4 ± 2.6	16.4 ± 1.3	<0.001
BMI (percentile)	72.1 ± 28.3	97.3 ± 1.3	46.5 ± 17.7	<0.001
WC (cm)	68.2 ± 12.5	78.5 ± 8.7	57.8 ± 4.9	< 0.001
Public school (%)	23.6	24.8	22.5	0.59

^a^Student’s *t*-test and Pearson’s *χ* ^2^ test. BMI, body mass index; WC, waist circumference.

**Table 2:** **Intake of energy and macronutrients by children, cases and controls**

**Daily nutrient intake**	**All**	**Obese**	**Normal-weight**	***p*** **-value** ^**a**^
	**(n = 402)**	**(n = 202)**	**(n = 200)**	
	**Median (25p, 75p)**	**Median (25p, 75p)**	**Median (25p, 75p)**	
Energy, (kcal/d)	2036 (1557, 2779)	1912 (1455, 2593)	2181 (1659, 2986)	0.002
% adequacy	109.2 (81.7, 144.1)	98.0 (75.0, 138.4)	114.5 (92.3, 151.9)	<0.001
Protein, (g/d)	73 (58, 100)	69 (55, 93)	77 (61, 110)	0.006
% kcal/d	14.5 (13.2, 16.1)	14.5 (13.3, 16.2)	14.6 (13.1, 15.9)	0.59
Carbohydrates, (g/d)	256 (183, 345)	243 (177, 322)	276 (192, 386)	0.003
% kcal/d	49.4 (45.1, 53.7)	49.5 (45.9, 54)	49.1 (44.9, 53.7)	0.85
Total lipids, (g/d)	83 (62, 118)	79 (60, 106)	90 (67, 128)	0.003
% kcal/d	37.3 (33.4, 40.8)	37.0 (33.4, 40.6)	37.6 (33.3, 41)	0.63
Saturated fats, (g/d)	30 (22, 42)	28 (21, 39)	32 (23, 45)	0.002
% kcal/d (totals)	13.3 (11.9, 14.7)	13.2 (11.8, 14.7)	13.5 (12, 14.8)	0.37
Polyunsaturated fats, (g/d)	15 (10, 21)	14 (9, 19)	16 (11, 24)	0.01
% kcal/d (totals)	6.3 (5.3, 7.6)	6.3 (5.4, 7.5)	6.3 (5.3, 7.8)	0.94
Total cholesterol, (mg/d)	264 (189, 355)	251 (175, 332)	282 (200, 376)	0.007
Fiber, (g/d)	15 (11, 21)	14 (10, 18)	16 (11, 22)	0.006
% adequacy	74.3 (52.3, 99.7)	69.3 (49.3, 92.5)	77.7 (58.5, 108.5)	0.003

^a^Mann-Whitney’s *U* test. p, percentile.

According to the reported food habits and the parents’ information, 51.8% of obese children vs 65.6% of normal-weight children had healthy dietary habits (*p* = 0.007) (Table 3).

**Table 3:** **Food habits, physical activity and sedentary lifestyles in cases and control children**

**Characteristics**	**All**	**Obese**	**Normal-weight**	***p*** **-value** ^**a**^
	**(%)**	**(%)**	**(%)**	
**Food habits**				
Parents information^b^ (n)	383	194	189	
Having healthy dietary habits^c^	58.6	51.8	65.6	0.007
Not having breakfast at home	23.5	26.5	20.3	0.15
Not bringing lunch to school	25.9	28.4	23.3	0.24
Bringing money to buy food at school	12.1	14.1	10.2	0.24
Children information^d^ (n)	402	202	200	
Having healthy dietary habits^c^	54.5	50.0	58.0	0.15
Not having breakfast at home	18.7	17.3	20.0	0.49
Not bringing lunch to school	18.4	22.3	14.5	0.04
Bringing money to buy food at school	34.3	36.1	32.5	0.44
**Physical activity**				
At school (h/week)^e^				
<1	67.7	72.8	62.5	0.02
≥1	32.3	27.2	37.5	
Outside of school (METs) (tertiles)^f^				
1 (0)	49.2	49.0	49.5	
2 (0.17-2.52)	17.4	17.8	17.0	
3 (2.53-14.28)	33.3	33.2	33.5	0.97
**Sedentary**				
Televisions at home (number)				
1-2	46.0	40.6	51.5	
3-4	54.0	59.4	48.5	0.02
Hours in front of the screen				
≤2	44.8	41.1	48.5	
>2	55.2	58.9	51.5	0.13
Transportation (home/school)				
Other	37.6	34.2	41.0	
Car	62.4	65.8	59.0	0.15
**Sleep hours**				
<9 h	39.3	44.5	33.4	
≥9 h	60.7	55.5	66.6	0.03

^a^Pearson’s *X* ^2^ test.

^b^Information given by parents covering the week prior to the interview.

^c^Healthy dietary habits defined as follows: eating breakfast at home, bringing lunch to school, and not bringing money to purchase food.

^d^Information given by children only about the day prior to the interview.

^e^Independent of the time dedicated to recreation for the children.

^f^MET, metabolic equivalent.

Obese children spent less time in physical activity than eutrophic children, as shown by physical activity in schools during the prior week (*p* = 0.02). In terms of household appliances that promote a sedentary lifestyle such as television sets, 59.4% of obese children had 3–4 in their households compared with only 48.5% of the households of normal-weight children (*p* = 0.02). A higher proportion of obese children slept less than 9 hours than eutrophic children (*p* = 0.03).

The association between obesity and dietary habits, exercise, and sedentary behavior is presented in Table 4. Children with healthy dietary habits exhibited a decreased risk of obesity compared with children with unhealthy dietary habits (OR: 0.59; 95% CI: 0.46:0.75). Regarding the consumption of carbohydrates without cardiovascular risk, the children in the highest tertile of fruit consumption exhibited a decreased risk for obesity (OR: 0.63; 95% CI: 0.42:0.95). By contrast, the high consumption of foods containing carbohydrates with cardiovascular risk, such as traditional foods with added fat (tertiles high vs. low; OR: 2.12; 95% CI: 1.37:3.27; *p Trend* = 0.002) and natural fruit juice (tertiles high vs. low; OR: 1.78; 95% CI: 1.10:2.90, *p Trend* = 0.04), were associated with an increased risk of obesity. In addition, consuming sweetened commercial beverages (tertiles high vs. low) was also associated with an increased risk of obesity (OR: 1.58; 95% CI: 0.94:2.67).

**Table 4:** **Association between the intake of carbohydrates, exercise and sedentary lifestyles and risk for obesity in schoolchildren**

**Characteristics**	**Model 1** ^**a**^	**Model 2** ^**b**^
	**OR (95% CI)**	**OR (95% CI)**
**Dietary habits** ^c^		
Unhealthy dietary habits	1	
Healthy dietary habits^d^	0.59 (0.46;0.75)	
**Foods containing carbohydrates without cardiovascular risk**		
Fruits (tertiles) (g/d)		
1 (0–47.7)	1	1
2 (50.0-102.8)	0.77 (0.46;1.31)	0.83 (0.49;1.40)
3 (114.3-600.0)	0.63 (0.42;0.95)	0.63 (0.42;0.94)
*p Trend*	0.02	0.01
Vegetables (tertiles) (g/d)		
1 (0–34.3)	1	1
2 (35.7-85.7)	0.39 (0.22;0.68)	0.29 (0.20;0.42)
3 (87.1-350.0)	0.85 (0.39;1.86)	0.77 (0.42;1.41)
*p Trend*	*0*.71	0.41
Cereals (tertiles) (g/d)		
1 (0–56.0)	1	1
2 (56.1-115.0)	0.99 (0.71;1.39)	1.20 (0.80;1.82)
3 (115.3-7 31.4)	1.14 (0.62;2.07)	1.44 (0.63;3.28)
*p Trend*	0.78	0.47
**Foods with carbohydrates with cardiovascular risk**		
Fried foods made of wheat or corn (tertiles) (g/d)^e^		
1 (0–35.1)	1	1
2 (35.7-82.3)	1.35 (1.05;1.75)	1.69 (1.14;2.50)
3 (82.4-410.8)	2.12 (1.37;3.27)	3.48 (1.72;7.02)
*p Trend*	0.002	0.002
Natural fruit juice (tertiles) (mL/d)		
1 (0–24.3)	1	1
2 (24.6-71.4)	0.82 (0.47;1.42)	0.78 (0.39;1.57)
3 (72.8-750)	1.78 (1.10;2.90)	2.16 (1.28;3.65)
*p Trend*	0.04	0.02
Sweetened commercial beverages (tertiles) (mL/d)		
1 (0–155.7)	1	1
2 (157.1-392.8)	1.75 (1.20;2.56)	1.91 (1.13;3.24)
3 (408.3-1939.3)	1.58 (0.94;2.67)	1.73 (1.07;2.79)
*p Trend*	0.09	0.04
**Physical activity**		
At school (h/week)^f^		
<1	1	1
≥1	0.33 (0.15;0.72)	0.37 (0.16;0.89)
**Sedentary behavior**		
Television sets at home (number)		
1-2	1	1
3-4	1.60 (0.97;2.66)	2.13 (1.20;3.78)
Transportation, school/home		
Other	1	
Car	1.35 (1.06;1.71)	
**Sleep hours**		
<9 h	1	1
≥9 h	0.65 (0.42;1.02)	0.64 (0.40;1.03)

^a^OR adjusted for age, sex and energy, and a correlation within schools was considered.

^b^Model adjusted for age, sex, energy, METs, and listed variables, correlation among schools was considered.

^c^Information provided by parents regarding the eating habits of their children during the week prior to the study.

^d^Eating breakfast at home, bringing lunch to school, and not bringing money to buy food at school.

^e^Traditional foods with added fat.

^f^Independent of the time dedicated to recreation by the children.

Regarding physical activity, the children who performed ≥1 h/wk of exercise at school demonstrated an inverse association for the risk of obesity compared with those who performed <1 h/wk (OR: 0.33; 95% CI: 0.15:0.72). The analysis of sedentary activities, assessed by the number of television sets at home, indicated that having 3–4 television sets at home and using the car to go at school were associated with an increased risk of childhood obesity. In addition, sleeping ≥9 hours was identified as a protective factor for obesity.

## Discussion

Due to the well-defined categories of nutritional status in the case (obese) and eutrophic (control) children, we hypothesized that the cases would consume more energy than the controls. However, the results showed that obese children consumed fewer calories (~270 Kcal) than eutrophic children, which alone does not explain why they are obese.

The children’s energy intake values were estimated using the FFQ, which has been widely validated for this purpose, and the values were consistent in both groups. Compared with the Mexican data, obese children had an adequacy intake ratio of 98%, while eutrophic children had an adequacy ratio of 114.5% [17]. Similar findings have been reported in two nutritional national surveys, in which the FFQ and 24-hour recalls were used [19,20]. In both surveys, obese children reportedly ingested fewer calories, a finding that was considered to be underreported. In another thorough study of girls, their reported energy intakes were categorized as plausible, under-reported or over-reported, and the participants who under-reported their energy intake had higher body fat [21]. Therefore, the first explanation of this paradox would be that there could have been under-reporting of food consumption, and procedures that would have made under-reporting unfeasible were not applied in our study.

Independent of the quantity of energy, some factors can help explain why the obese children were obese. First, concerning eating habits, parents’ information about the week prior to the survey showed that obese children ate breakfast at home less frequently than eutrophic children; in addition, obese children were less likely to bring lunch to school and took money to buy food at school more frequently. Concerning these unhealthy eating habits of obese children, other studies have shown that children who skip breakfast experience deregulation of their appetites and metabolic changes. As a result, food bought at school is consumed when extremely hungry and eventually leads to abdominal obesity and insulin resistance [22].

When examining the intake of foods and beverages, this report shows that obese children are more greatly exposed to cardiovascular risk foods than eutrophic children. Additionally, the dietary fiber intake of both normal-weight and obese children was barely more than half the recommended intake; however, a higher percentage of obese children consumed less fiber than normal-weight children. This finding suggests that foods consumed by obese children might be more industrialized and refined. On the other hand, obese children consume more foods typical of Mexico than eutrophic children, such as *tamales, sopes,* and *tacos* made of corn or wheat and vegetable oil or animal fat, which were associated with an increased risk of obesity. Other studies have reported that the consumption of these foods by children already starts at preschool ages [23].

Likewise, our data show that obese children consume more natural juice or sweetened commercial beverages, such as soft drinks, than eutrophic children, which only provide carbohydrates, especially fructose. These drinks do not lead to glycogen synthesis but result in sustained elevations in postprandial triglyceride levels [24].

This study revealed that obese children performed fewer physical activities at school than eutrophic children. This finding, combined with the inferred sedentary habits of these obese children due to having more television sets at home and commuting more frequently to school, might explain the differences in nutritional statuses between normal-weight and obese children. Moreover, these children’s habits are associated with the lifestyles found in cities with large metropolitan areas, which are designed for cars instead of people; thus, such children do not have much space for outdoor play. Furthermore, issues of security and violence may preclude recreational activities for children in public spaces [25]; consequently, parents often provide sedentary entertainment to compensate for the lack of these activities [26].

Our results highlight two consequences of obesity. First, we found that the obese children were taller than the normal-weight children of similar ages, which might have resulted from accelerated bone maturation due to an imbalance in the growth hormone axis (GHA) involving insulin-like growth factor 1 (IGF-1). This process may have occurred during the prepubertal period but may not necessarily lead to a greater final height in these children [27,28]. Additionally, this study confirmed that obese children slept fewer hours than normal-weight children, and these alterations have also been shown to be associated with abnormal glucose metabolism, increased pro-inflammatory cytokines, and eating disorders, which are risk factors for developing childhood obesity [29,30].

Compared with obese children, eutrophic children showed healthier habits. On the other hand, the higher intake of calories by normal-weight children compared with obese children may involve genetic factors that either predispose or protect against obesity. Previously published results of this project have found a relationship between the rs12255372 variant of the *TCF7L2* gene and obesity; however, this gene is associated with diseases such as T2D in adults, but it appears to protect against the development of obesity in children [9].

Among its weaknesses, the present study was cross-sectional; the eating and exercise/sedentary habits, both in school and at home, were gathered using questionnaires that were validated. However, no other procedures were used to confirm that the information about food intake was under- or over-reported.

However, many of the present findings are confirmatory, and the strength of the present study was the simultaneous analysis of dietary habits, exercise level and sedentary behavior in children and the classification in advance by nutritional status of children who were either of a normal weight or obese.

## Conclusions

Despite fewer calories ingested by obese children versus normal-weight children, their nutritional status can be explained by bad habits, such as skipping breakfast, not bringing lunch to school and bringing money to buy food at school. Additionally, differences in nutritional status could be explained by the type of food and drink consumed because the results indicate that obese children consume more fatty foods and sugary drinks than eutrophic children. In addition, obese children performed fewer physical activities at school, slept fewer hours and had more sedentary routines.

This study shows that obesity in school children has a number of complex determinants, driven by the acquisition of habits that could either be risky or beneficial to their health. However, an obesogenic environment could be ameliorated if teachers and parents work together to form healthy habits such as not skipping breakfast. Similarly, scientific information can be translated to strengthen the capacity of parents and students to choose healthy foods and beverages as well as to encourage physical activity and discourage sedentary behaviors.
